# Prey‐Foraging Patterns in a Complex Landscape of Fear

**DOI:** 10.1002/ece3.73405

**Published:** 2026-04-08

**Authors:** Monika Sysiak, Barbara Pietrzak, Andrzej Mikulski

**Affiliations:** ^1^ Department of Hydrobiology, Institute of Ecology, Faculty of Biology, Biological and Chemical Research Centre University of Warsaw Warsaw Poland

## Abstract

Prey antipredator defenses may weaken when a predator is cannibalistic or stressed, but it remains unclear which predator prey systems show this effect and how strongly it shapes trophic cascades. In our aquatic food chain (higher order predator, mesopredator, and mesopredator's prey), we found no defense suppression in mesopredators exposed to chemical cues signaling higher order predator cannibalism or stress; instead, their defensive responses increased, resulting in distinct and cue‐specific hunting patterns with varied consequences for their prey. These results suggest that suppression is system‐specific and highlight the value of network‐based behavioral analysis for revealing subtle shifts in hunting behavior.

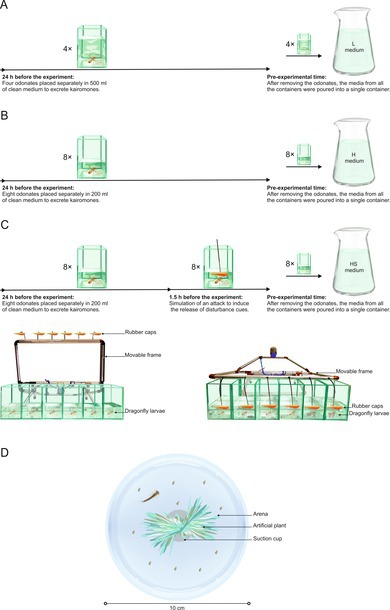

## 1 Introduction

One of the fundamental responses to predators is for organisms to adopt defensive strategies, which can involve changes to morphology, physiology, behavior or life history (Werner and Peacor 2003). However, implementing such strategies almost always comes at a significant cost. Therefore, it is extremely important that the prey's response is matched to the actual pressure of the predator as overestimating the risk may mean incurring unnecessary costs. This matching is known in the literature as a threat‐sensitive response (Helfman 1989), and has been studied in various groups of animals (Bursztyka et al. 2018; Dure Ruiz et al. 2018; Vavrek and Brown 2009; Webb et al. 2009). The threat assessment in prey is usually based on stimuli perceived from the environment, such as visual (Helfman 1989) and chemical cues (Wagner et al. 2023). The latter play a particularly important role in aquatic environments, where their reliability is enhanced by the persistence of infochemical gradients in water, which has a higher viscosity compared to the more turbulent air (Pohnert et al. 2007). Among infochemicals, kairomones appear to be the most prevalent carriers of information regarding predation risk (Kats and Dill 1998). They benefit the receiver rather than the emitter, by definition (Brown Jr et al. 1970). The ability of potential prey to detect kairomones released by predators and adjust their traits accordingly may enhance their ability to avoid predation (De Meester et al. 1999).

However, threat perception is not limited to the detection of predator kairomones. The interpretation of these cues, and therefore the strength and nature of the induced defense response, may depend on additional contextual information that determines the severity of the threat. For instance, *Daphnia* exhibit defensive responses to kairomones released by visually oriented predators when exposed to light. However, these responses are suppressed in the dark, reflecting the reduced hunting efficiency of predators without light. This plasticity allows *Daphnia* to respond adaptively to fluctuating environmental conditions while minimizing the energetic costs of defense (Effertz and Von Elert 2014).

Prey threat assessment may also depend on the strength of the cues, i.e., the concentration of kairomones, which corresponds to predator density. Most studies indicate that an increase in the concentration of these chemical cues is associated with a stronger prey response. Yet, this relationship may vary depending on the system. Sysiak, Maszczyk, and Mikulski (2025) showed that high kairomone concentrations can signal safety to prey when cannibalism is common within the predator population. As the intensity of cannibalism usually increases with population density (Rosenheim and Schreiber 2022), high concentrations of kairomones imply a greater likelihood of cannibalism. Therefore, when individuals in cannibalistic populations detect high concentrations of chemical cues from conspecifics, they may perceive them as cues of food availability or potential danger (Moir and Weissburg 2009; Tran 2014). Whether they position themselves as cannibals or prey in response to these cues is irrelevant. In both cases, they are less motivated to prey on other species. This mechanism is supported by previous research showing that damselfly larvae decrease their hunting activity when exposed to high concentrations of conspecific cues (Sysiak et al. 2023). Therefore, in the case of prey of a different species in such systems, an increase in kairomone concentration may not necessarily indicate an increase in predation risk, but rather a decrease.

Quantitative information about the level of threat may also be conveyed through cues reflecting the predator's motivational state. For example, prey can use chemical cues to determine a predator's diet (Brown and Dreier 2002; Chivers et al. 1996; Laurila et al. 1997) or hunger level and adjust their anti‐predator responses accordingly. This pattern has been observed in both vertebrate and invertebrate predator–prey systems (Bell et al. 2006; Smith and Belk 2001). A predator's motivational state may also be influenced by its stress level when exposed to threats from higher‐order predators. However, much less is known about how prey respond to chemical cues indicating that their predators may themselves be under threat. Such cues may include alarm cues released by injured individuals as well as those originating from their digestion by the predator (Laforsch et al. 2006; Stabell et al. 2003), and disturbance cues emitted by stressed individuals that have been approached by a predator but were not injured. Conspecifics interpret both types of cues as indicators of danger and may activate defensive mechanisms in response (Crane et al. 2022). Sysiak, Maszczyk, and Mikulski (2025) showed that in *Daphnia* exposure to such predator‐derived alarm or disturbance cues can suppress defensive responses normally triggered by predator kairomones.

Previous studies have shown that prey can use cues indicating predator stress or high densities of cannibalistic predators to avoid triggering unnecessary defenses (Sysiak, Maszczyk, and Mikulski 2025). However, the functional framework of this mechanism remains unclear. Prey species generally evolve either specialized defenses finely tuned to specific predators or generalist defenses that provide moderate protection against multiple predator types, which often reflects the nature of predation pressure in their environment (Brown and Robinson 2016; McPeek et al. 1996; Van Tienderen 1991). This type of defense suppression appears to be a specialized response to specific predators rather than a general one. It depends on specific traits of the predator, such as cannibalistic tendencies or occupying a mid‐trophic position, associated with vulnerability to higher‐order predators. The key feature of the prey, in turn, is that it must constitute an important part of such a predator's diet and thus belong to the corresponding trophic level. Therefore, testing these effects in different predator–prey systems could provide a more comprehensive understanding of the functional aspects of these mechanisms and reveal which prey traits make species vulnerable to mechanisms. Moreover, examining such systems at the upper levels of the trophic cascade, specifically by studying cues from higher‐order predators and the corresponding responses of mesopredators, would show if these effects can propagate to lower trophic levels and how far‐reaching their consequences are for trophic cascade dynamics.

To better understand prey defense suppression under conditions of predator cannibalism or stress, we quantified the hunting behavior of an aquatic mesopredator, the *Ischnura elegans* damselfly larvae, when exposed to different quantities and qualities of chemical cues released by a higher‐order predator, the *Sympetrum sanguineum* dragonfly larvae. We then evaluated how these induced behavioral changes influenced hunting success and, consequently, the mesopredator *I. elegans* pressure exerted on their *Daphnia* prey. Both odonates are common and widespread, frequently co‐occurring (Buczyński et al. 2022; Moore 1991). Damselfly larvae are known for their behavioral flexibility in response to predator‐derived chemical cues (Brown and Robinson 2016; Sysiak et al. 2023). Dragonfly larvae, in turn, may themselves fall prey to higher‐order predators such as fish and are also known for their cannibalistic tendencies (Pierce 1988; Van Buskirk 1989). These characteristics make both species a suitable predator–prey model for investigating mechanisms of defense suppression.

Since previous studies revealed inconsistent recognition of predator cannibalism‐related cues by prey (Sysiak, Maszczyk, and Mikulski 2025), we propose two possible scenarios of response to the same cues combinations.

In the first scenario, where *S. sanguineum* cannibalism plays a minor role for *I. elegans* , we hypothesized that: (1) the strength of the defensive response of *I. elegans* to kairomones increases proportionally with *S. sanguineum* kairomone concentration, (2) but when *I. elegans* are simultaneously exposed to high concentrations of kairomones and *S. sanguineum* disturbance cues, their defensive response is suppressed.

In the second, representing the cannibalistic *S. sanguineum* scenario, we hypothesized that: (1) *I. elegans* shows a defensive response at low concentrations of kairomones from a cannibalistic predator, (2) but at high concentrations this response is suppressed, (3) and disturbance cues further enhance this suppression.

## 2 Materials and Methods

### 2.1 Experimental Animals

#### 2.1.1 Higher Order Predator

Larvae of the dragonfly *Sympetrum sanguineum* were collected in June 2021 from a small pond in Warsaw (52°07′49.8″ N, 21°02′53.1″ E), a natural urban pond inhabited by both fish and invertebrate predators. As these higher‐order predators served as a source of chemical cues, several procedures were implemented to standardize the individuals and minimize variability in cue production. After being transferred to the laboratory, the larvae were handled individually and assigned identification codes. Larvae were photographed and measured using NIS‐Elements BR 3.2 software and classified into size classes according to the method described by Sysiak, Baczyński, and Mikulski (2025). Individuals with a head width of 3.48–3.75 mm and a total body length of 8.24–9.82 mm were selected for cue production because these mid‐stage individuals can prey on *I. elegans* larvae in the 10th instar, while also being potential prey for larger conspecifics or other predators. All larvae were kept individually in conditioned (aerated for at least 2 weeks) and filtered (1 μm filter) lake water from the city pond Glinianki Szczęśliwickie in Warsaw, Poland (52°12′25.1″ N, 20°57′36.1″ E), and fed Chironomidae larvae every other day.

#### 2.1.2 Mesopredator

The focal animals were damselfly larvae *Ischnura elegans* collected in July 2021 from Lake Czerniakowskie in Warsaw, Poland (52°11′16.4″ N, 21°04′28.3″ E), a natural urban lake inhabited by both fish and invertebrate predators—including *S. sanguineum* (due to the structure of the littoral zone, *S. sanguineum* was easier to collect from another reservoir), and transferred to the laboratory. They were then synchronized to the 10th instar as this stage was selected as large enough to facilitate observations of their behavior in recordings. The larvae were photographed and measured using NIS‐Elements BR 3.2 software and assigned to instars based on the parameters proposed by Thompson (1978): body length and head width. Instar assignments were additionally confirmed by visual inspection of the stage of wing bud development. The 9th instar individuals were reared in a specially designed system as described by Sysiak et al. (2023). They were fed live adult *Daphnia magna* every other day. Upon reaching the 10th instar, the larvae were transferred to a thermostatic chamber at 4°C to inhibit further molting (Shepard and Lutz 1976) and each placed in a separate container to prevent cannibalistic interactions. Knowing the date of the larvae's last molt allowed us to design the experiment to avoid unexpected molting during exposure to kairomones. In addition, as injury may affect larval behavior (Gyssels and Stoks 2005), all focal individuals were examined for signs of mechanical damage (e.g., loss of lamellae or legs) and excluded if any was found. All larvae were kept in conditioned lake water (as described above).

#### 2.1.3 Prey to Mesopredator

The water flea *Daphnia magna* was obtained from a clonal culture established from hatched ephippial eggs obtained from Nový Vrbenský Rybník, České Budějovice, Czech Republic (49°00′33.4″ N, 14°26′40.9″ E) and maintained in the laboratory of the Department of Hydrobiology UW since 2014.

### 2.2 Media Preparation

**FIGURE 1:**
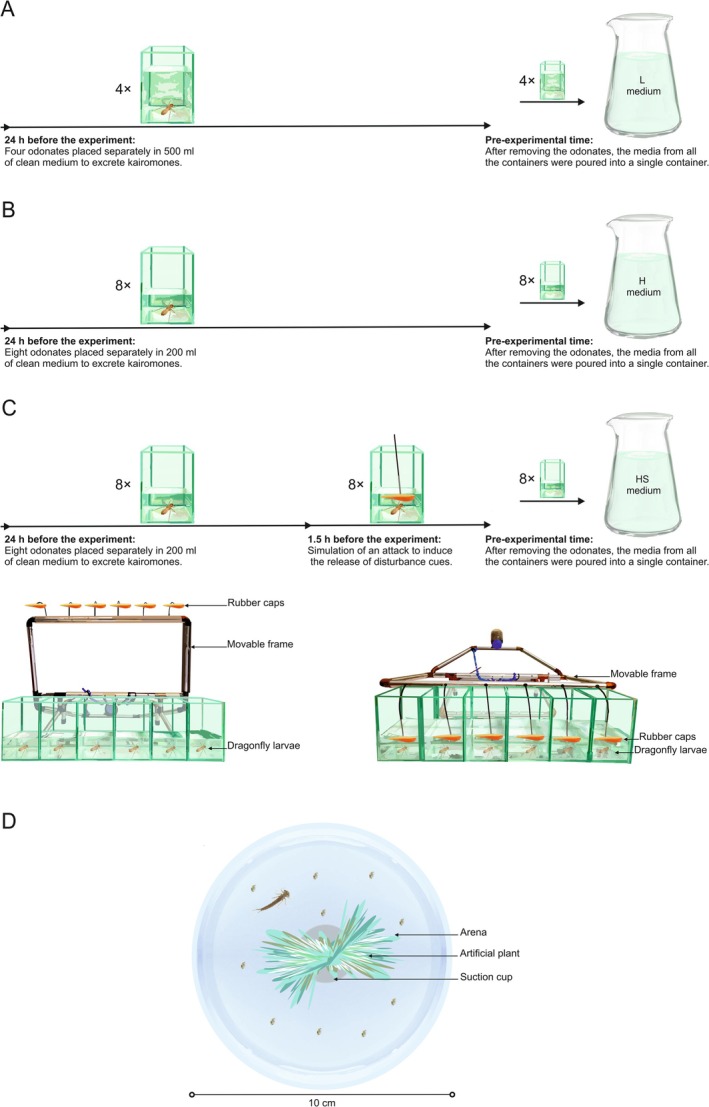
Experimental media preparation (A) L: Kairomone from 2 *S. sanguineum* × L^−1^, (B) H: Kairomone from 5 *S. sanguineum* × L^−1^, (C) HS: Similar to H but with additional cue from stressed *S. sanguineum* . The arrows represent timelines of experimental media preparation. Glass vessels containing larvae indicate low (A) and high (B) volumes, corresponding to low and high concentrations of kairomone cues, respectively. The numbers next to the vessels indicate replicates. (C) Contains an extra vessel labeled 1.5 h before experiment, marking the subsequent step necessary for HS medium preparation above this timeline at left. Disturbance installation consisting of a movable frame with streamlined rubber caps attached to it, shown in its “before scaring” position. Square glass containers, each containing 200 mL of lake water and one dragonfly larva, were placed under this installation. At right Disturbance installation, in its “during scaring” position. The movable frame has been lowered, causing the streamlined rubber caps to plunge violently into the water of containers holding dragonfly larvae, triggering their stress and escape behavior. (D) An individual experimental container with arena zone and refuge zone (artificial plant on suction cup) with the focal mesopredator—damselfly larva—and their *Daphnia* prey released.

To simulate different population densities and motivational states of the higher order predator, we used two concentrations of kairomones, based on previous studies showing that these particular concentrations of conspecific kairomones influenced the responses of *S. sanguineum* (Sysiak, Baczyński, and Mikulski 2025). The media used in the experiments were as follows: C: control medium (without any cues); L: medium with information on low density of higher order predators (two *S. sanguineum* larvae per liter) (Figure 1A); H: medium with information on high density of higher order predators (five *S. sanguineum* larvae per liter) (Figure 1B); HS: medium with information on high density of higher order predators and higher order predator disturbance cues (chemicals released by stressed predators) (Figure 1C).

The control medium (C) contained only lake water, filtered through a 1 μm polypropylene fiber filter and then aerated for 2 weeks. The same water was used in the remaining media. Media L and H were prepared by placing starved (to prevent contamination by alarm cues) *S. sanguineum* larvae each separately in a vessel filled either with 500 mL or 200 mL of water, respectively, 24 h before the experiments. To create media with different higher order predator densities we used single individuals exposed in different volumes of water rather than varying the number of individuals in the same volume (Figure 1A,B). We took this approach to eliminate any competitive or cannibalistic interactions that might otherwise arise between individuals. *S. sanguineum* larvae were not fed for 2 days prior to media preparation to avoid contaminating their chemical cues with food items that could potentially serve as a source of prey alarm cues (Ferrari et al. 2010). The medium HS was prepared similarly to medium H (Figure 1B,C), with the addition that the disturbance cues were generated by stressing *S. sanguineum* in each vessel for 90 min before the experiment. The stress was induced mechanically and visually by automatically lowering a frame with rods ending in streamlined rubber caps, causing the caps to plunge violently into the water every 10 min, simulating attacks (Figure 1C). Similar to previous studies (Sysiak, Baczyński, and Mikulski 2025), to minimize variability resulting from potential unequal cue release among individual larvae, media from all vessels used for a given treatment were filtered through a GFC filter immediately before the experiment and pooled into a single container.

### 2.3 Experimental Procedure

The experiment took place in a specially designed chamber with stable lighting (11.1 ± 0.3 μmol m^−2^ s^−1^, measured with a Li‐COR 250A photometer), a temperature of 20°C and equipped with a camera (setup detailed by Sysiak et al. (2023)). Six days before the experiment, the mesopredators were acclimatized by transferring them from 4°C to 20°C. At this time, each mesopredator was individually placed in a round, opaque plastic vessel (10 cm diameter, 3 cm height) filled with 200 mL of control medium and containing a centrally located refuge zone consisting of an artificial plant attached with a 2 cm silicone suction cup (Figure 1D). Each tested mesopredator was initially fed 10 pre‐reproductive stage *D. magna* . The live food was simultaneously introduced into the vessels through a feeding device. Three days before the experiment, the medium was refreshed and the mesopredators were fed as before. The mesopredators were then starved for 2 days before the day of the experiment.

On the day of the experiment, each vessel was filled with 200 mL of one of the experimental media. The experimental unit was the larva. Treatments C and L included eight larvae each, while treatments H and HS included seven larvae each for a total of 30 larvae. The mesopredators were randomly assigned to the treatments and then allowed a 30 min acclimatization period to reduce any stress effects of the pre‐experimental procedures on their behavior. After acclimatization, they were simultaneously provided with 10 *Daphnia* each, and the recording of hunting behavior started, which lasted for 63 min.

### 2.4 Recording Analysis

The first 3 min of the raw recordings were removed as the mesopredators may have shown altered behavior due to disturbances caused by the feeding device and the closing of the set‐up. The following 15 min were analyzed, as hunting activity typically decreases over time, even if the hunt is unsuccessful (Bell 1990). We created a separate timeline for each individual, and nine exclusive behaviors were manually identified by the observer based on the video recordings. Each was coded with three or two letters. The first letter denotes where the behavior takes place, in refuge (R) or in arena (A). The second letter denotes whether the animal is immobile (I) or mobile (M). The third letter specifies further either the location in refuge: on plant (P) or on cup (C), or the type of mobility: leaning (L), treading (T), gentle movements (G), or swimming (S). The behaviors were: (1) RIP: Immobility on plant—most camouflaging behavior; (2) RIC: Immobility on suction cup—observation of prey; (3) RMP: Mobility on plant (vertical movement)—stalking of prey; (4) RMC: Mobility on suction cup (horizontal fast walking)—stalking of prey; (5) RML: Leaning out in search of prey—stalking of prey; (6) AI: Immobility in arena—observation of prey; (7) AMG: Gentle movements in arena (turning the head behind the prey, slow initial walking)—initial stalking of prey; (8) AMT: Treading in arena (decisive, quick following of the prey)—main stalking of prey; (9) AMS: Swimming.

### 2.5 Behavioral Parameters

To investigate mesopredator threat sensitivity response, we used a network analysis (Batool and Niazi 2014), where each observed behavior is a node and the directed edges of the network represent transitions between behaviors. This approach differs from classical behavioral analyses, such as measuring time spent in a shelter, which is typically interpreted as a foraging reduction strategy (Pierce 1988), but which overlooks the possibility that foraging may still occur within the shelter, obscuring information about the threat sensitivity response. The network analysis provided a detailed insight into behavioral patterns by identifying key behaviors and sequences of foraging actions.

Using this approach, we calculated the following parameters: (i) the total number of transitions between behaviors, and (ii) degree centrality (DC) of behavior, which indicates how often a behavior occurs and how strongly it is connected with other behaviors, thus reflecting its role in behavioral sequences, including those related to hunting. It was calculated as:

$$\mathrm{DC}\left(V\right)=\frac{k_{v}}{n-1}$$

where: *k*_*v*_—the total number of links to a behavior, *n*—the total number of behaviors (Batool and Niazi 2014). We also recorded (iii) total consumption—the number of consumed *Daphnia*.

### 2.6 Statistical Analysis

All analyses were performed using the R environment ver. 4.4.3 (R Core Team 2024). Data distribution was assessed using the Shapiro–Wilk test for normality, skewness, kurtosis, and Levene's test for homogeneity of variance (Hardin and Hilbe 2007).

To assess the effect of the experimental treatments on the total number of transitions we used a generalized linear model (GLM) with a negative binomial (NB) distribution fitted with the glm.nb function from the MASS package, as this distribution is suitable for modeling count data with zeros (Hardin and Hilbe 2007). Model adequacy was evaluated through examination of deviance residuals, including Quantile–Quantile (QQ) plots and residuals vs. predicted values plots.

To analyze the effect of experimental treatments on degree centrality of behaviors we used a generalized linear mixed model (GLMM) with a Tweedie distribution and a log link (Dunn and Smyth 2018). We analyzed eight behaviors, excluding RML, which was only observed in isolated cases and consisted primarily of zeros, making it unsuitable for analysis. We fitted the model using the glmmTMB package. Using cell‐means parameterization, we estimated a mean for each treatment behavior combination without defining a reference level. A random intercept for each larva accounted for repeated measurements within individuals. Larval identity was defined as a unique identifier across the entire dataset (Appendix S2). The Tweedie GLMM is particularly suitable for modeling continuous outcomes derived from counting processes (Dunn and Smyth 2018). The adequacy of the model was tested using DHARMa simulation diagnostics, including checks of the residual distribution, dispersion, zero‐inflation, and outliers. Inference focused on estimated marginal means (EMMs) and prespecified contrasts rather than main effects, which are undefined under the cell‐means parameterization. EMMs were obtained using the emmeans package. The following comparisons were conducted: (i) between treatment comparisons within each behavior, and (ii) between behavior comparisons within each treatment. We report z‐tests with Holm adjustment for multiplicity.

To evaluate the influence of the experimental treatments on the number of consumed *Daphnia* we used a GLM model with a Poisson distribution. Model adequacy was evaluated using dispersion statistics (i.e., Pearson residuals relative to degrees of freedom) and a chi‐squared goodness‐of‐fit test. We inspected model residuals using plots of deviance residuals versus predicted values and QQ plots.

To test the relationship between hunting success and behavioral changes induced by chemical cues, we used GLM with a quasi‐Poisson distribution and a log link (Hardin and Hilbe 2007). The dependent variable was the number of consumed *Daphnia*, and each model included a behavioral parameter (total number of transitions or degree centrality of individual behaviors), the experimental treatment and their combinations. This allowed us to assess both the overall effect of behavioral parameters on hunting success and whether this relationship differed between treatments. We chose the quasi‐Poisson to account for the overdispersion commonly observed in count data. We fitted a separate model for each behavioral variable and extracted the corresponding parameter estimates, *t*‐values, and *p*‐values. We evaluated model adequacy using pseudo‐*R* ^2^ and an overdispersion index. Additionally, we inspected diagnostic plots of Pearson residuals versus fitted values to assess variance homogeneity and potential systematic deviations.

## 3 Results

Overall, most of the mesopredators (80%) spent over half of the analyzed time immobile in the refuge (RI) and less than 10% of the time moving in the arena (AM).

### 3.1 Total Number of Transitions

**FIGURE 2:**
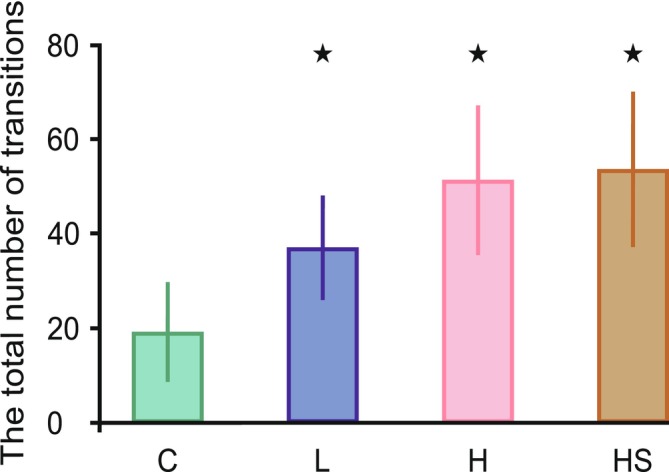
Total number of transitions between behaviors of damselfly *I. elegans* larvae exposed to: C = control, different concentrations of dragonfly kairomones L = low, H = high, and HS = high kairomones + disturbance cues. The bars represent the mean and the whiskers standard deviation. Star symbols indicate treatments that are significantly different from the control.

The NB GLM analysis for the total number of transitions showed significant differences between the treatments. Compared to the control (C), all experimental treatments: low kairomone (L), high kairomone (H), and high kairomone with disturbance cues (HS) showed a statistically significant increase in the total number of transitions between behaviors (Figure 2, Appendix S1).

### 3.2 Degree Centrality of Behaviors

**FIGURE 3:**
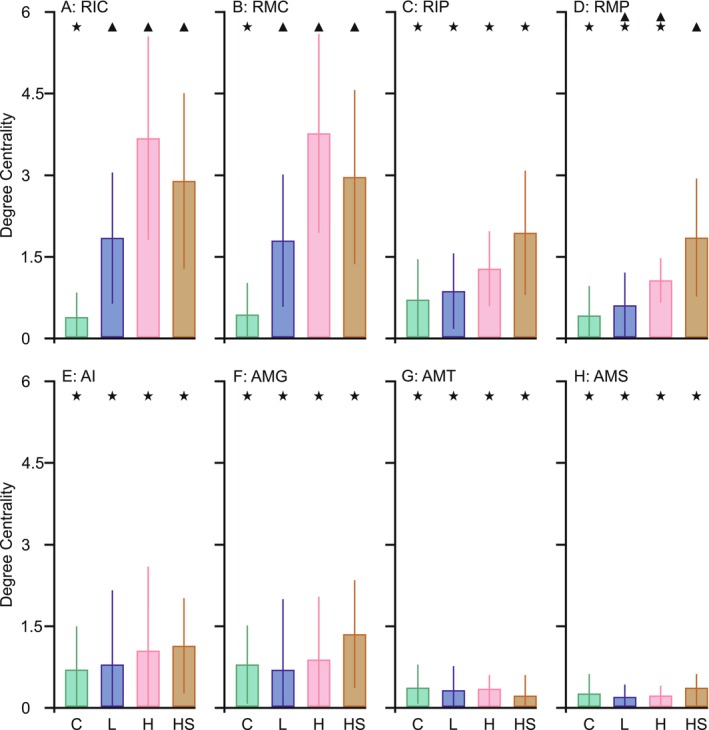
Between‐treatment comparisons of degree centrality for eight behaviors. Each panel shows a distinct behavior: (A) RIC (immobility on suction cup), (B) RMC (mobility on suction cup), (C) RIP (immobility on plant), (D) RMP (mobility on plant), (E) AI (immobility in arena), (F) AMG (gentle movements in arena), (G) AMT (treading in arena), and (H) AMS (swimming). Treatments included: C = control, different concentrations of dragonfly kairomones L = low, H = HS = high kairomones + disturbance cues. The bars represent the mean and the whiskers standard deviation. Distinct symbols (star, circle, triangle) indicate significant differences between treatments.

Pairwise contrasts of estimated marginal means (EMMs) from the Tweedie GLMM with a cell‐means parameterization revealed clear between‐treatment differences for degree centrality of immobility on suction cup (RIC) and mobility on suction cup (RMC). In both behaviors, all predator‐cue treatments (L, H, and HS) showed higher degree centrality than control (C). For mobility on plant (RMP), HS exhibited higher centrality than C. For the remaining behaviors, between‐treatment differences were not significant (Figure 3, Appendix S3).

**FIGURE 4:**
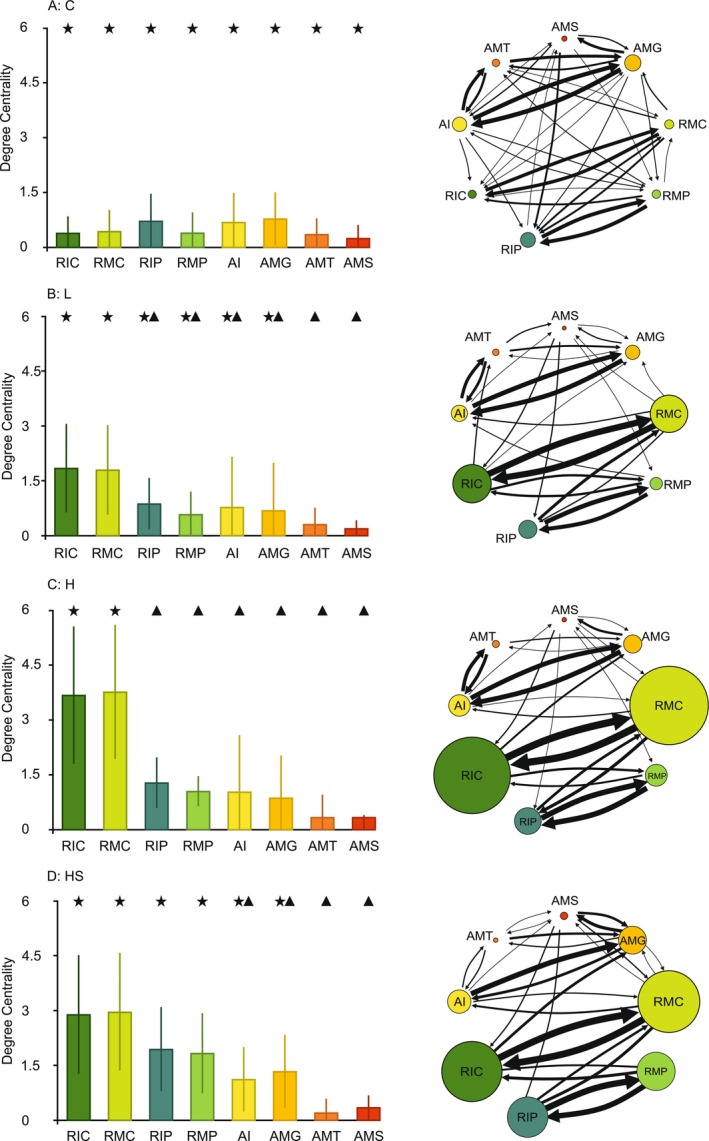
Between‐behavior comparisons of degree centrality for: RIC (immobility on suction cup), RMC (mobility on suction cup), RIP (immobility on plant), RMP (mobility on plant), AI (immobility in arena), AMG (gentle movements in arena), AMT (treading in arena), and AMS (swimming), across treatments. Each panel shows a distinct treatment: (A) C = control, (B) L = low kairomones, (C) H = high kairomones, (D) HS = high kairomones + disturbance cue. The bars represent the mean and the whiskers standard deviation. Distinct symbols (star, triangle) indicate significant differences between behaviors. Network diagrams to the right of each bar graph illustrate hunting patterns: Arrow thickness corresponds to log (number of transitions in the given direction) × 2, while node size represents degree centrality (DC × 6) of each behavior.

Within‐treatment comparisons of degree centrality showed no significant differences between behaviors in treatment C (Figure 4A). Treatment L displayed higher degree centrality for RIC and RMC than for treading in an arena (AMT) and swimming (AMS) (Figure 4B). In treatment H, RIC and RMC had degree centrality significantly higher than all other behaviors (Figure 4C). In treatment HS, all behaviors in refuge had higher centrality than AMT and AMS (Figure 4D).

All contrast results are presented in Appendix S4.

### 3.3 Daphnia Consumption

The Poisson GLM analysis for the *Daphnia* consumption showed no significant differences between control (C) and the other treatments (L, H, HS) (Appendix S5).

### 3.4 Influence of Changes in Behavior on Hunting Success

Quasi‐Poisson GLM models revealed significant differences in how interactions between total number of transitions, degree centrality of arena treading (AMT), and swimming (AMS) and experimental treatment influenced *Daphnia* consumption.

**FIGURE 5:**
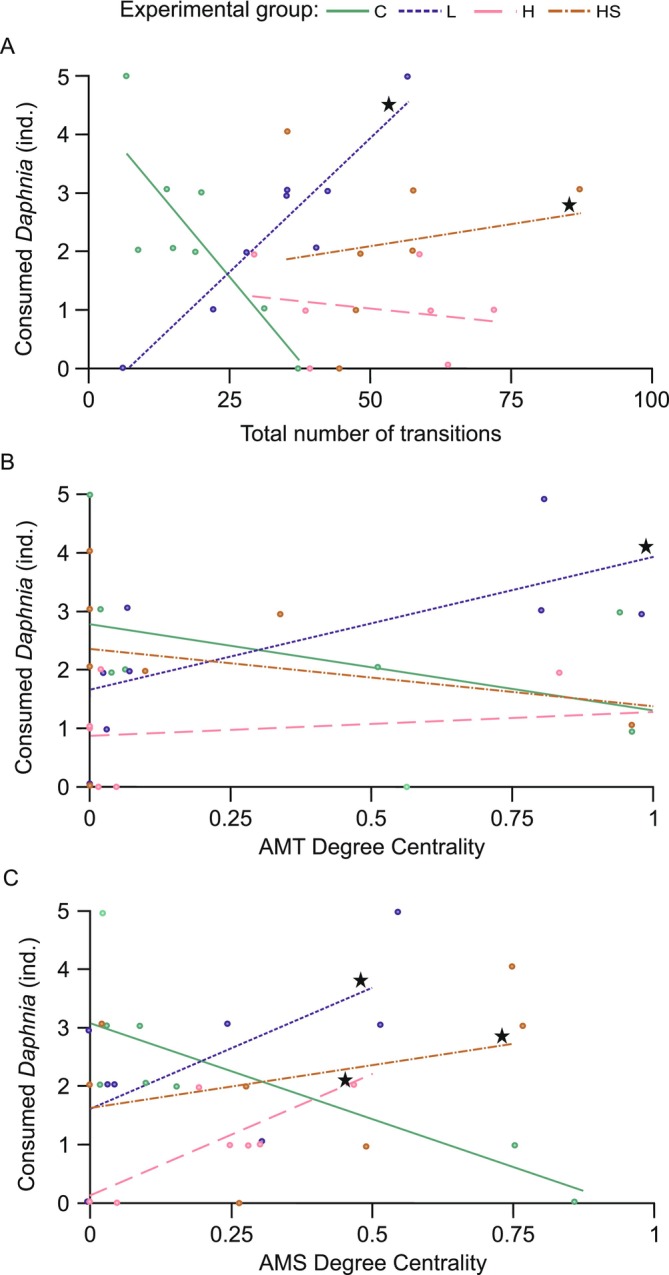
Relationship between behavioral differences and hunting success: The effect of the interaction between behavioral parameters of *I. elegans* larvae—(A) total number of transitions, (B) degree centrality of treading in the arena (AMT), and (C) degree centrality of swimming (AMS)—and C = control, different concentrations of kairomones L = low kairomones, H = high kairomones, and HS = kairomones + disturbance cues on *Daphnia* consumption. To improve the visibility of individual data points, jitter (0.06) has been applied to both the horizontal and vertical axes. Star symbols indicate significant interaction effect compared to control.

In the control treatment (C) an increased total number of transitions was negatively associated with *Daphnia* consumption. Conversely, a significantly different trend was observed in the L and HS treatments, where more transitions were associated with increased *Daphnia* consumption. In treatment H, changes in the total number of transitions did not affect *Daphnia* consumption (Figure 5A).

Higher AMT degree centrality was linked to reduced *Daphnia* consumption in the control treatment. Conversely, a significantly different trend was observed in the L treatment: increased AMT degree centrality was associated with higher *Daphnia* consumption. In treatment H and HS, changes in degree centrality of AMT did not affect *Daphnia* consumption (Figure 5B).

In the control treatment, higher AMS degree centrality was associated with lower consumption. Conversely, a significantly different trend was observed in all treatments with chemical cues (L, H, and HS): increased AMS degree centrality was associated with higher *Daphnia* consumption (Figure 5C).

Full model results are presented in Appendix S6.

## 4 Discussion

The results obtained seem to partially confirm the first scenario proposed: damselfly larvae exhibited a stronger threat response at higher densities of predatory dragonfly larvae, which suggests that predator cannibalism is not a significant factor for these particular larvae. However, the results fit only partially. Contrary to the hypothesis, disturbance cues did not suppress this effect. Instead, they appeared to enhance it.

Compared to the control treatment, larvae from all treatments with chemical cues exhibited a higher degree centrality of refuge behaviors. In other words, they intensified the transformations into and between refuge behaviors, making them the most common choice for the larvae. The high degree centrality of these behaviors also suggests that they may have constituted the core of the hunting strategy, as they were most strongly connected with the rest of the behavioral repertoire. Consequently, other behaviors may have been more likely to be initiated through them. Thus, perceived predator pressure caused hunting to shift to the refuge, which is a common anti‐predator response (Dionne et al. 1990; Sysiak et al. 2023). However, the location of these behaviors varied depending on the specific chemical cues. Both in the low (L) and high (H) kairomone concentration treatments, the behaviors were concentrated on the suction cup. In contrast, in the high kairomone concentration with disturbance cues (HS) treatment, key behaviors occurred not only on the suction cup but also on the plant, which provided greater hiding potential than the suction cup (Figure 3).

Moreover, we identified behavioral parameters associated with intensifying hunting success in each treatment (Figure 5). This together with the observed disproportion between behaviors within each treatment (Figure 4) led us to define four distinct hunting patterns:

1. Control (C) pattern was characterized by relatively few total transitions (Figure 2) between behaviors, none of which stood out in terms of centrality (Figure 4A), suggesting that each behavior played a similar role in hunting. Greater behavioral diversity with fewer transitions indicates longer, more evenly distributed time spent on each behavior. This pattern appeared to be optimal in terms of hunting success. Deviations from this, such as an increase in total transitions (Figure 5A) or a greater reliance on arena swimming (AMS) (Figure 5C), led to reduced consumption by control individuals.
2. Low kairomone (L) pattern was characterized by a significant within‐ treatment disproportion: refuge behaviors such as observing prey from a safe place (immobility on suction cup—RIC) or adjusting position relative to the target (mobility on suction cup—RMC), showed higher degree centrality compared to the more dynamic hunting behaviors in the arena, such as quickly following prey (AMT) or swimming (AMS). This disproportion was driven by an increase in RIC and RMC degree centrality, while for AMT and AMS it stayed similar to that in the control treatment (Figure 3). Consequently, the overall rise in the total number of transitions in this treatment (71% higher than in the control) (Figure 2; Appendix S1) was most likely invested by the larvae in transitions between RIC (immobility) and RMC (mobility) on the suction cup. Such frequent switching likely results in shorter durations of these behaviors, so distances on the suction cup were covered by several short moves with pauses rather than by single long moves. This pattern may help mesopredators avoid detection by a visually hunting higher order predator (Heads 1985; Jeffries 1990). On the other hand, this pattern may be due to the refuge design. A centrally located refuge enabled the damselfly to maintain visual contact with its prey while making only minor, subtle movements around the suction cup. In contrast, tracking prey from elsewhere in the container would have required longer and more conspicuous movements (Figure 1D). The observation that an increased number of transitions improved hunting success in this treatment (Figure 5A) suggests that this movement style is an adjustment to hunting conditions in the refuge rather than a strategy to avoid visual predators. Nevertheless, refuge hunting was not an optimal strategy overall. Because the refuge area was smaller than the arena, damselflies had fewer opportunities to encounter *Daphnia* (Figure 1D). This is supported by the observation that hunting success increased with greater activity outside the refuge, particularly through the more frequent use of arena treading (AMT) and swimming (AMS) (Figure 5B,C). Hypothetically, these rapid arena behaviors may reflect a safer strategy: quick capture of *Daphnia* and swift return to the refuge. We often observed this scenario with AMT and AMS use by larvae. However, both arena treading and swimming were rare (Figure 4B). Therefore, low concentrations of *S. sanguineum* kairomone overall inhibit the hunting opportunities of *I. elegans* .
3. High kairomone (H) pattern appears to be a more intense version of the low kairomone pattern, as RIC and RMC centrality increased more strongly, differing significantly from all other refuge and arena behaviors (Figure 4C). The number of transitions also rose by 169% compared to controls (Figure 2; Appendix S1), but this no longer translated into more prey captures, likely due to limited prey number in the small refuge (Figure 5A). Instead, hunting success depended on arena activity, primarily swimming (Figure 5C).
4. High kairomone + disturbance cues (HS): RIC and RMC remained key central behaviors, but these conditions also promoted a greater reliance on camouflage strategies: immobility and mobility on the plant (RIP, RMP). In contrast, arena treading (AMT) and swimming (AMS) were rare compared to all refuge behaviors (Figure 4D). This more diverse use of refuge space is consistent with Wellborn and Robinson (1987), who reported that odonate larvae classified plant structures by their camouflage or foraging potential, selecting them according to factors such as hunger level or predator pressure. In our setup, this classification likely applied not only to the suction cup and plant distinction but also to different parts of the plants themselves. Thus, damselflies may also have used plant related behaviors for hunting, as suggested by the positive association between increased transitions and prey capture (Figure 5A). They made 180% more transitions than the control treatment (Figure 2; Appendix S1). Unlike in other treatments, these transitions were distributed across all four refuge behaviors (Figure 4D), indicating that these individuals exploited the three‐dimensional refuge space more effectively than in the other treatments. Even so, hunting within the refuge was not generally the most successful strategy, since prey capture increased with swimming in the arena (Figure 5C). Thus, the stronger investment in developing a refuge hunting, where prey availability is limited, may indicate that kairomone with disturbance cues elicited the strongest predator avoiding compared to the other treatments.

Despite these different hunting strategies, we observed no differences between treatments in the final number of *D. magna* consumed (Appendix S5). The costs of predator exposure may initially manifest at other levels of individual functioning, for example through the initiation of physiological processes (Jiang et al. 2019; Sysiak et al. 2023) preparing the organism for defense or potential injuries following a predator attack (Balderrama et al. 1992; Pettersen et al. 2020; Murray et al. 2020). Under such conditions, maintaining rather than reducing resource intake may help offset the increased energetic demands. However, over longer observation periods, differences in overall prey consumption could emerge. Restricting foraging to a limited area may gradually reduce local prey availability. Importantly, the treatment‐specific relationships between behavioral parameters and hunting success (Figure 5) suggest that certain behaviors are beneficial or detrimental to hunting success. These patterns indicate that sustained reliance on refuge‐centered strategies could ultimately reduce overall prey intake.

The intensification of predator avoidance by prey in response to increasing predator densities is a commonly observed phenomenon (Brown and Dreier 2002; Chivers et al. 1996; Laurila et al. 1997). Thus, suppression of prey defense upon information about predator cannibalism is likely reserved for unique predator–prey interactions. The simplest explanation for the lack of suppression in our studies is that cannibalism does not significantly affect the population of higher order predators. However, this seems unlikely. Previous studies on *S. sanguineum* larvae have shown that their metabolism changes when exposed to increased kairomone concentrations from conspecifics (Sysiak, Baczyński, and Mikulski 2025). However, in populations where cannibalism is common, these cues may indicate either a threat or food availability, triggering defensive behavior or increased prey searching. Thus, an increase in metabolic activity alone only signals interest; it does not fully explain motivation. Chandra et al. (2006) showed that higher densities of conspecifics in Aeshnidae can intensify predation activity on mosquito larvae, most likely as a way of countering competitors. Yet, Aeshnidae generally exhibit more active behavior than the Libellulidae species used in our study (Pritchard 1965).

Another possible explanation for the absence of prey defense suppression is that *I. elegans* from this habitat are not adapted to respond specifically to this particular predator. The pond from where they originated contains diverse predators, including fish. Damselflies adapt their responses to local predation pressure (Gyssels and Stoks 2006, 2005), and cope with predator diversity by specializing in defenses against a specific predator or by developing general anti‐predator strategies through flexible phenotypic expression (Brown and Robinson 2016; McPeek et al. 1996; Van Tienderen 1991). Therefore, to determine whether predator cannibalism suppresses defensive behaviors in *I. elegans* , further experiments should compare the responses of individuals originating from ponds with different predator pressure.

An interesting result of our study is that *I. elegans* exhibited the strongest defensive response to disturbance cues compared to the other treatments. This contrasts with studies on *Daphnia*, which exhibit defense suppression in response to predator disturbance cues (Sysiak, Maszczyk, and Mikulski 2025). Our results are similar to those observed in *Ischnura cervula* , which increased feeding activity when exposed to conspecific disturbance cues (Siepielski et al. 2016). There is a gap in the literature regarding how individuals respond to disturbance cues released by other species, especially the response of prey to predator disturbance cues. In our study, *I. elegans* may interpret *S. sanguineum* disturbance cues as a threat due to shared predators or the close relationship between damselflies and dragonflies, which makes these signals less species‐specific (Crane et al. 2022).

In this study, we show that the hypothesis of prey defense suppression when predators are cannibalistic or stressed likely applies only to specific predator–prey interactions and requires further verification. Our results indicate that damselflies exhibit stronger defenses under high concentrations of dragonfly kairomones and disturbance cues. However, our detailed behavioral analysis provides insight into shifts in prey hunting strategies under the threat, offering a useful tool in ecological research to capture subtle differences. We also provide insights into the role of predator disturbance cues, showing that when predator and prey are closely related, the cues may convey the same information to both prey and predator.

## Author Contributions

**Monika Sysiak:** conceptualization (equal), data curation (lead), formal analysis (lead), funding acquisition (lead), investigation (lead), methodology (equal), project administration (lead), resources (equal), software (lead), visualization (lead), writing – original draft (lead), writing – review and editing (equal). **Barbara Pietrzak:** formal analysis (equal), validation (equal), writing – review and editing (lead). **Andrzej Mikulski:** conceptualization (equal), data curation (equal), methodology (equal), resources (equal), supervision (lead), validation (equal), writing – review and editing (equal).

## Funding

This work was supported by the National Science Centre, Poland (2018/31/N/NZ8/03800).

## Conflicts of Interest

The authors declare no conflicts of interest.

## Supporting information


**Appendix S1:** Results of the NB GLM model examining the effects of chemical cues (groups) on the total number of transitions.
**Appendix S2:** Structure of the generalized linear mixed model (GLMM) used to analyze degree centrality of behaviors.
**Appendix S3:** Pairwise comparisons of experimental groups within each behavior. Results are based on estimated marginal means from the Tweedie GLMM, with z‐tests adjusted for multiple comparisons using the Holm method.
**Appendix S4:** Pairwise comparisons of DC of behaviors within each experimental group (C, L, H, HS). Results are based on estimated marginal means from the Tweedie GLMM, with z‐tests adjusted for multiple comparisons using the Holm method.
**Appendix S5:** Results of the Poisson GLM model examining the effects of chemical cues (groups) on the number of *Daphnia* consumed.
**Appendix S6:** Results of quasi‐Poisson GLM models examining the interaction effect of behavioral parameters and experimental group on the number of *Daphnia* consumed.

## Data Availability

Data are available as supporting information: https://figshare.com/articles/dataset/Z‐A/29093375.
