# Supplementary material for: Prey‐Foraging Patterns in a Complex Landscape of Fear
Source: Ecol Evol. 2026 Apr 8;16(4):e73405. doi: 10.1002/ece3.73405 (PMC13059677; doi:10.1002/ece3.73405)
Supplement: Supplementary file 1 — Appendix S1: Results of the NB GLM model examining the effects of chemical cues (groups) on the total number of transitions. Appendix S2: Structure of the generalized linear mixed model (GLMM) used to analyze degree centrality of behaviors. Appendix S3: Pairwise comparisons of experimental groups within each behavior. Results are based on estimated marginal means from the Tweedie GLMM, with z‐tests adjusted for multiple comparisons using the Holm method. Appendix S4: Pairwise comparisons of DC of behaviors within each experimental group (C, L, H, HS). Results are based on estimated marginal means from the Tweedie GLMM, with z‐tests adjusted for multiple comparisons using the Holm method. Appendix S5: Results of the Poisson GLM model examining the effects of chemical cues (groups) on the number of Daphnia consumed. Appendix S6: Results of quasi‐Poisson GLM models examining the interaction effect of behavioral parameters and experimental group on the number of Daphnia consumed. [file ECE3-16-e73405-s001.docx]

**Appendix S1**

Results of the NB GLM model examining the effects of chemical cues (groups) on the total number of transitions.

| **Group** | **Coefficient (Estimate)** | **Standard Error** |  | **t-value** | **p-value** |
| --- | --- | --- | --- | --- | --- |
| Intercept | 2.94 | 0.15 |  | 19.22 | **<0.001** |
| L | 0.54 | 0.21 |  | 2.61 | **<0.001** |
| H | 0.99 | 0.21 |  | 4.64 | **<0.001** |
| HS | 1.03 | 0.21 |  | 4.85 | **<0.001** |

**Appendix S2**

Structure of the generalized linear mixed model (GLMM) used to analyze degree centrality of behaviors.

| **Model component** | **Specification** |
| --- | --- |
| Distribution / link | Tweedie / log |
| Fixed effects | *Treatment × behavior* |
| Random effect | Larva ID (intercept) |
| Variance (Larva ID) | 0.0897 |
| SD (Larva ID) | 0.299 |
| Number of individuals | 30 |
| Observations | 240 |

**Appendix S3**

Pairwise comparisons of experimental groups within each behavior. Results are based on estimated marginal means from the Tweedie GLMM , with z-tests adjusted for multiple comparisons using the Holm method.

| **DC of behavior** | **Contrast** | **Estimate** | **Standard Error** | **z-value** | **p-value** |
| --- | --- | --- | --- | --- | --- |
| RIC | C * L | -1.549 | 0.505 | -3.068 | **0.008** |
|  | C * H | -2.271 | 0.488 | -4.649 | **<0.001** |
|  | C * HS | -2.022 | 0.496 | -4.076 | **<0.001** |
|  | L*H | 0.722 | 0.341 | 2.117 | 0.102 |
|  | L*HS | 0.473 | 0.352 | 1.345 | 0.357 |
|  | H*HS | 0.249 | 0.327 | 0.76 | 0.447 |
| RMC | C * L | -1.422 | 0.493 | -2.885 | **0.015** |
|  | C * H | -2.19 | 0.474 | -4.029 | **<0.001** |
|  | C * HS | -1.943 | 0.482 | -4.029 | **<0.001** |
|  | L*H | 0.768 | 0.341 | 2.25 | 0.073 |
|  | L*HS | 0.521 | 0.352 | 1.479 | 0.278 |
|  | H*HS | 0.247 | 0.325 | 0.761 | 0.446 |
| RIP | C * L | -0.202 | 0.477 | -0.424 | 0.921 |
|  | C * H | -0.592 | 0.465 | -1.274 | 0.811 |
|  | C * HS | -1.009 | 0.441 | -2.289 | 0.132 |
|  | L*H | 0.39 | 0.447 | 0.874 | 0.921 |
|  | L*HS | 0.807 | 0.422 | 1.912 | 0.279 |
|  | H*HS | -0.417 | 0.408 | -1.021 | 0.921 |
| RMP | C * L | -0.38 | 0.569 | -0.668 | 0.589 |
|  | C * H | -0.991 | 0.539 | -1.838 | 0.264 |
|  | C * HS | -1.542 | 0.509 | -3.029 | **0.014** |
|  | L*H | 0.611 | 0.497 | 1.229 | 0.589 |
|  | L*HS | 1.161 | 0.464 | 2.504 | 0.061 |
|  | H*HS | -0.55 | 0.426 | -1.292 | 0.589 |
| AI | C * L | -0.059 | 0.5 | -0.817 | 1 |
|  | C * H | -0.399 | 0.489 | -0.817 | 1 |
|  | C * HS | -0.506 | 0.481 | -1.052 | 1 |
|  | L*H | 0.34 | 0.485 | 0.702 | 1 |
|  | L*HS | 0.447 | 0.477 | 0.937 | 1 |
|  | H*HS | -0.106 | 0.465 | -0.229 | 1 |
| AMG | C * L | 0.198 | 0.5 | 0.395 | 1 |
|  | C * H | -0.113 | 0.491 | -0.231 | 1 |
|  | C * HS | -0.549 | 0.458 | -1.2 | 1 |
|  | L*H | 0.311 | 0.508 | 0.612 | 1 |
|  | L*HS | 0.747 | 0.477 | 1.566 | 0.703 |
|  | H*HS | -0.436 | 0.466 | -0.935 | 1 |
| AMT | C * L | 0.068 | 0.668 | 0.102 | 1 |
|  | C * H | 0.168 | 0.668 | 0.256 | 1 |
|  | C * HS | 0.498 | 0.732 | 0.68 | 1 |
|  | L*H | 0.1 | 0.687 | 0.145 | 1 |
|  | L*HS | -0.33 | 0.748 | -0.441 | 1 |
|  | H*HS | 0.43 | 0.765 | 0.565 | 1 |
| AMS | C * L | 0.281 | 0.769 | 0.365 | 1 |
|  | C * H | 0.078 | 0.77 | 0.102 | 1 |
|  | C * HS | -0.418 | 0.703 | -0.595 | 1 |
|  | L*H | 0.202 | 0.806 | 0.251 | 1 |
|  | L*HS | 0.699 | 0.743 | 0.94 | 1 |
|  | H*HS | -0.497 | 0.734 | -0.669 | 1 |

**Appendix S4**

Pairwise comparisons of DC of behaviors within each experimental group (C, L, H, HS). Results are based on estimated marginal means from the Tweedie GLMM, with z-tests adjusted for multiple comparisons using the Holm method.

| **Group** | **Contrast** | **Estimate** | **Standard Error** | **z-value** | **p-value** |
| --- | --- | --- | --- | --- | --- |
| C | RIC*RMC | -0.102 | 0.583 | -0.176 | 1 |
|  | RIC*RIP | -0.626 | 0.536 | -1.167 | 1 |
|  | RIC*RMP | -0.037 | 0.591 | -0.063 | 1 |
|  | RIC*AI | 0.567 | 0.541 | 1.047 | **1** |
|  | RIC*AMG | 0.695 | 0.531 | 1.309 | 1 |
|  | RIC*AMT | -0.096 | 0.606 | -0.158 | 1 |
|  | RIC*AMS | -0.483 | 0.657 | -0.734 | 1 |
|  | RMC*RIP | 0.523 | 0.524 | 0.998 | 1 |
|  | RMC*RMP | 0.065 | 0.58 | 0.112 | 1 |
|  | RMC*AI | 0.464 | 0.529 | 0.8977 | 1 |
|  | RMC*AMG | -0.593 | 0.519 | 1.143 | 1 |
|  | RMC*AMT | -0.198 | 0.595 | -0.333 | 1 |
|  | RMC*AMS | -0.585 | 0.647 | -0.904 | 1 |
|  | RIP*RMP | 0.588 | 0.531 | 1.107 | 1 |
|  | RIP*AI | -0.059 | 0.476 | -0.124 | 1 |
|  | RIP*AMG | 0.069 | 0.465 | 0.149 | 1 |
|  | RIP*AMT | -0.721 | 0.549 | -1.314 | 1 |
|  | RIP*AMS | -1.109 | 0.605 | -1.833 | 1 |
|  | RMP*AI | 0.529 | 0.537 | 0.985 | 1 |
|  | RMP*AMG | 0.658 | 0.527 | 1.249 | 1 |
|  | RMP*AMT | -0.133 | 0.603 | -0.221 | 1 |
|  | RMP*AMS | -0. 52 | 0.654 | -0.796 | 1 |
|  | AI*AMG | -0.129 | 0.469 | -0.274 | 1 |
|  | AI*AMT | 0.662 | 0.553 | 1.199 | 1 |
|  | AI*AMS | 1.049 | 0.609 | 1.724 | 1 |
|  | AMG*AMT | 0.791 | 0.543 | 1.457 | 1 |
|  | AMG*AMS | 1.178 | 0.6 | 1.964 | 1 |
|  | AMT*AMS | -0.387 | 0.667 | -0.58 | 1 |
| L | RIC*RMC | 0.024 | 0.332 | 0.073 | 1 |
|  | RIC*RIP | 0.721 | 0.386 | 1.867 | 0.867 |
|  | RIC*RMP | 1.131 | 0.428 | 2.642 | 0.197 |
|  | RIC*AI | -0.923 | 0.407 | -2.27 | 0.44 |
|  | RIC*AMG | -1.052 | 0.42 | -2.503 | 0.271 |
|  | RIC*AMT | -1.812 | 0.519 | -3.49 | **0.012** |
|  | RIC*AMS | -2.312 | 0.606 | -3.813 | **0.003** |
|  | RMC*RIP | -0.696 | 0.387 | -1.797 | 0.939 |
|  | RMC*RMP | 1.107 | 0.429 | 2.578 | 0.228 |
|  | RMC*AI | -0.899 | 0.408 | -2.203 | 0.496 |
|  | RMC*AMG | -1.027 | 0.421 | -2.437 | 0.295 |
|  | RMC*AMT | -1.788 | 0.52 | -3.436 | **0.014** |
|  | RMC*AMS | -2.288 | 0.607 | -3.767 | **0.004** |
|  | RIP*RMP | 0.41 | 0.472 | 0.87 | 1 |
|  | RIP*AI | -0.202 | 0.453 | -0.447 | 1 |
|  | RIP*AMG | -0.331 | 0.466 | -0.711 | 1 |
|  | RIP*AMT | -1.092 | 0.556 | -1.962 | 0.796 |
|  | RIP*AMS | -1.591 | 0.639 | -2.492 | 0.271 |
|  | RMP*AI | 0.208 | 0.489 | 0.425 | 1 |
|  | RMP*AMG | 0.079 | 0.5 | 0.159 | 1 |
|  | RMP*AMT | -0.681 | 0.586 | -1.163 | 1 |
|  | RMP*AMS | -1.181 | 0.664 | -1.778 | 0.939 |
|  | AI*AMG | 0.129 | 0.48 | 0.268 | 1 |
|  | AI*AMT | 0.889 | 0.57 | 1.561 | 1 |
|  | AI*AMS | 1.389 | 0.65 | 2.137 | 0.554 |
|  | AMG*AMT | 0.761 | 0.58 | 1.312 | 1 |
|  | AMG*AMS | 1.261 | 0.659 | 1.914 | 0.835 |
|  | AMT*AMS | -0.5 | 0.726 | -0.689 | 1 |
| H | RIC*RMC | -0.022 | 0.27 | -0.082 | 1 |
|  | RIC*RIP | 1.053 | 0.344 | 3.063 | **0.037** |
|  | RIC*RMP | 1.242 | 0.361 | 3.438 | **0.011** |
|  | RIC*AI | -1.305 | 0.369 | -3.539 | **0.008** |
|  | RIC*AMG | -1.462 | 0.385 | -3.799 | **0.003** |
|  | RIC*AMT | -2.434 | 0.518 | -4.698 | **<0.001** |
|  | RIC*AMS | -2.832 | 0.59 | -4.798 | **<0.001** |
|  | RMC*RIP | -1.075 | 0.343 | -3.135 | **0.031** |
|  | RMC*RMP | 1.264 | 0.36 | 3.507 | **0.009** |
|  | RMC*AI | -1.327 | 0.368 | -3.608 | **0.006** |
|  | RMC*AMG | -1.484 | 0.384 | -3.865 | **0.002** |
|  | RMC*AMT | -2.456 | 0.518 | -4.746 | **<0.001** |
|  | RMC*AMS | -2.854 | 0.59 | -4.839 | **<0.001** |
|  | RIP*RMP | 0.189 | 0.418 | 0.453 | 1 |
|  | RIP*AI | -0.252 | 0.424 | -0.594 | 1 |
|  | RIP*AMG | -0.41 | 0.439 | -0.934 | 1 |
|  | RIP*AMT | -1.382 | 0.559 | -2.471 | 0.188 |
|  | RIP*AMS | -1.779 | 0.627 | -2.839 | 0.072 |
|  | RMP*AI | -0.063 | 0.439 | -0.143 | 1 |
|  | RMP*AMG | -0.22 | 0.453 | -0.486 | 1 |
|  | RMP*AMT | -1.192 | 0.571 | -2.09 | 0.421 |
|  | RMP*AMS | -1.591 | 0.637 | -2.498 | 0.187 |
|  | AI*AMG | 0.157 | 0.457 | 0.345 | 1 |
|  | AI*AMT | 1.13 | 0.573 | 1.97 | 0.488 |
|  | AI*AMS | 1.527 | 0.641 | 2.384 | 0.222 |
|  | AMG*AMT | 0.972 | 0.584 | 1.664 | 0.865 |
|  | AMG*AMS | 1.37 | 0.65 | 2.107 | 0.421 |
|  | AMT*AMS | -0.397 | 0.737 | -0.539 | 1 |
| HS | RIC*RMC | -0.023 | 0.296 | -0.079 | 1 |
|  | RIC*RIP | 0.387 | 0.322 | 1.202 | 1 |
|  | RIC*RMP | 0.443 | 0.326 | 1.359 | 1 |
|  | RIC*AI | -0.95 | 0.368 | -2.583 | 0.167 |
|  | RIC*AMG | -0.778 | 0.352 | -2.108 | 0.381 |
|  | RIC*AMT | -2.615 | 0.604 | -4.333 | **<0.001** |
|  | RIC*AMS | -2.086 | 0.509 | -4.103 | **<0.001** |
|  | RMC*RIP | -0.411 | 0.321 | -1.279 | 1 |
|  | RMC*RMP | 0.466 | 0.325 | 1.435 | 1 |
|  | RMC*AI | -0.973 | 0.367 | -2.654 | 0.151 |
|  | RMC*AMG | -0.801 | 0.351 | -2.282 | 0.337 |
|  | RMC*AMT | -2.639 | 0.603 | -4.377 | **<0.001** |
|  | RMC*AMS | -2.11 | 0.508 | -4.155 | **<0.001** |
|  | RIP*RMP | 0.056 | 0.348 | 0.16 | 1 |
|  | RIP*AI | -0.562 | 0.388 | -1.45 | 1 |
|  | RIP*AMG | -0.391 | 0.373 | -1.047 | 1 |
|  | RIP*AMT | -2.228 | 0.616 | -3.617 | **0.007** |
|  | RIP*AMS | -1.699 | 0.523 | -3.1248 | **0.025** |
|  | RMP*AI | -0.506 | 0.391 | -1.295 | 1 |
|  | RMP*AMG | -0.335 | 0.377 | -0.889 | 1 |
|  | RMP*AMT | -2.172 | 0.618 | -3.515 | **0.01** |
|  | RMP*AMS | -1.643 | 0.526 | -3.127 | **0.037** |
|  | AI*AMG | -0.172 | 0.413 | -0.416 | 1 |
|  | AI*AMT | 1.666 | 0.64 | 2.601 | 0.167 |
|  | AI*AMS | 1.137 | 0.552 | 2.059 | 0.513 |
|  | AMG*AMT | 1.837 | 0.623 | 2.908 | 0.072 |
|  | AMG*AMS | 1.309 | 0.542 | 2.414 | 0.252 |
|  | AMT*AMS | 0.529 | 0.731 | 0.724 | 1 |

**Appendix S5**

Results of the Poisson GLM model examining the effects of chemical cues (groups) on the number of *Daphnia* consumed.

| **Group** | **Coefficient (Estimate)** | **Standard Error** |  | **z-value** | **p-value** |
| --- | --- | --- | --- | --- | --- |
| Intercept | 0.811 | 0.235 |  | 3.440 | **<0.001** |
| L | 0.054 | 0.328 |  | 0.164 | 0.869 |
| H | -0.811 | 0.445 |  | -1.821 | 0.068 |
| HS | -0.048 | 0.349 |  | - 0.140 | 0.889 |

**Appendix S6**

Results of quasi-Poisson GLM models examining the interaction effect of behavioral parameters and experimental group on the number of *Daphnia* consumed.

| **Model** | **Group** | **Estimate** | **t–value** | **p-value** |
| --- | --- | --- | --- | --- |
| *Daphnia* ~ Transitions * Group | Intercept | 1.902 | 4.772 | **<0.001** |
|  | Transitions | -0.067 | -2.663 | **0.014** |
|  | L | -2.713 | -3.692 | **0.001** |
|  | H | -1.397 | -1.269 | 0.218 |
|  | HS | -1.51 | -1.835 | 0.08 |
|  | Transitions*L | 0.112 | 3.868 | **<0.001** |
|  | Transitions*H | 0.057 | 1.767 | 0.091 |
|  | Transitions*HS | 0.074 | 2.625 | **0.015** |
| *Daphnia* ~ RIC * Group | Intercept | 0.968 | 3.148 | **0.004** |
|  | RIC | -0.444 | -0.72 | 0.479 |
|  | L | -0.629 | -1.061 | 0.3 |
|  | H | 0.067 | 0.072 | 0.944 |
|  | HS | -0.324 | -0.498 | 0.624 |
|  | RIC*L | 0.706 | 1.085 | 0.289 |
|  | RIC*H | 0.126 | 0.187 | 0.853 |
|  | RIC*HS | 0.484 | 0.757 | 0.457 |
| *Daphnia* ~ RMC * Group | Intercept | 0.979 | 3.353 | **0.002** |
|  | RMC | -0.444 | -0.86 | 0.399 |
|  | L | -0.574 | -1.015 | 0.321 |
|  | H | 0.024 | 0.027 | 0.979 |
|  | HS | -0.4 | -0.606 | 0.551 |
|  | RMC*L | 0.68 | 1.226 | 0.233 |
|  | RMC*H | 0.146 | 0.254 | 0.802 |
|  | RMC*HS | 0.505 | 0.927 | 0.364 |
| *Daphnia* ~ RIP * Group | Intercept | 1.12 | 3.82 | **<0.001** |
|  | RIP | -0.52 | -1.399 | 0.176 |
|  | L | -0.204 | 0.43 | 0.671 |
|  | H | -0.889 | -1.005 | 0.326 |
|  | HS | -0.576 | -0.961 | 0.347 |
|  | RIP*L | 0.456 | 0.893 | 0.381 |
|  | RIP*H | 0.332 | 0.462 | 0.649 |
|  | RIP*HS | 0.627 | 1.45 | 0.161 |
| *Daphnia* ~ RMP * Group | Intercept | 1.098 | 4.104 | **<0.001** |
|  | RMP | -0.93 | -1.496 | 0.149 |
|  | L | -0.345 | -0.806 | 0.429 |
|  | H | -0.422 | -0.455 | 0.654 |
|  | HS | -0.43 | -0.74 | 0.467 |
|  | RMP*L | 1.11 | 1.518 | 0.143 |
|  | RMP*H | 0.256 | 0.242 | 0.811 |
|  | RMP*HS | 0.98 | 1.472 | 0.155 |
| *Daphnia* ~ AI * Group | Intercept | 0.916 | 3.168 | **<0.001** |
|  | AI | -0.163 | -0.517 | 0.61 |
|  | L | -0.339 | -0.851 | 0.404 |
|  | H | -1.184 | -2.099 | **0.047** |
|  | HS | -0.039 | -0.079 | 0.937 |
|  | AI*L | 0.437 | 1.283 | 0.213 |
|  | AI*H | 0.374 | 0.971 | 0.342 |
|  | AI*HS | 0.058 | 0.132 | 0.896 |
| *Daphnia* ~ AMG * Group | Intercept | 0.926 | 2.828 | **0.009** |
|  | AMG | -0.154 | -0.452 | 0.656 |
|  | L | -0.316 | -0.744 | 0.465 |
|  | H | -1.346 | -2.134 | **0.044** |
|  | HS | -0.128 | -0.239 | 0.813 |
|  | AMG*L | 0.427 | 1.165 | 0.256 |
|  | AMG*H | 0.532 | 1.171 | 0.254 |
|  | AMG*HS | 0.127 | 0.294 | 0.772 |
| *Daphnia* ~ AMT * Group | Intercept | 1.032 | 3.876 | **<0.001** |
|  | AMT | -0.723 | -1.193 | 0.245 |
|  | L | -0.518 | -1.26 | 0.221 |
|  | H | -1.174 | -2.312 | **0.03** |
|  | HS | -0.17 | -0.44 | 0.659 |
|  | AMT*L | 1.617 | 2.077 | **0.049** |
|  | AMT*H | 1.077 | 1.309 | 0.204 |
|  | AMT*HS | 0.177 | 0.176 | 0.862 |
| *Daphnia* ~ AMS * Group | Intercept | 1.19 | 5.703 | **<0.001** |
|  | AMS | -2.467 | -2.323 | **0.029** |
|  | L | -0.701 | -1.982 | 0.06 |
|  | H | -2.317 | -3.255 | **0.003** |
|  | HS | -0.691 | -1.693 | 0.105 |
|  | AMS*L | 4.139 | 3.055 | **0.005** |
|  | AMS*H | 6.669 | 3.058 \| | **0.005** |
|  | AMS*HS | 3.149 | 2.488 | **0.02** |
|  |  |  |  |  |
